# “*Living like an empty gas tank with a leak*”: Mixed methods study on post-acute sequelae of COVID-19

**DOI:** 10.1371/journal.pone.0279684

**Published:** 2022-12-30

**Authors:** Oluwabunmi Ogungbe, Sarah Slone, Abeer Alharthi, Tosin Tomiwa, Baridosia Kumbe, Alanna Bergman, Katherine McNabb, Rhonda Smith Wright, Jason E. Farley, Cheryl R. Dennison Himmelfarb, Lisa A. Cooper, Wendy S. Post, Patricia M. Davidson, Yvonne Commodore-Mensah

**Affiliations:** 1 School of Nursing, Johns Hopkins University, Baltimore, Maryland, United States of America; 2 Texas Tech University, Lubbock, Texas, United States of America; 3 Center for Infectious Disease and Nursing Innovation, School of Nursing, Johns Hopkins University, Baltimore, Maryland, United States of America; 4 School of Medicine, Johns Hopkins University, Baltimore, Maryland, United States of America; 5 Bloomberg School of Public Health, Johns Hopkins University, Baltimore, Maryland, United States of America; 6 University of Wollongong, Wollongong, Australia; Food and Drug Administration, UNITED STATES

## Abstract

**Background:**

The burden and presentation of post-acute sequela of SARS-CoV-2 infection (PASC) are a developing major public health concern.

**Objectives:**

To characterize the burden of PASC in community-dwelling individuals and understand the experiences of people living with PASC.

**Methods:**

This mixed-methods study of COVID-19 positive community-dwelling persons involved surveys and in-depth interviews. Main outcome was self-report of possible PASC symptoms 3 weeks or longer after positive COVID-19 test. In-depth interviews were guided by a semi-structured interview guide with open-ended questions and probes based on emerging literature on PASC and the impact of COVID-19.

**Results:**

With a survey response rate of 70%, 442 participants were included in this analysis, mean (SD) age 45.4 (16.2) years, 71% female, 12% Black/African American. Compared to those with no PASC symptoms, persons who reported PASC symptoms were more likely to be older (mean age: 46.5 vs. 42; *p = 0*.*013)*, female (74.3% vs. 61.2%; *p = 0*.*010*), to have pre-existing conditions (49.6% vs. 34%; *p = 0*.*005*), and to have been hospitalized for COVID-19 (14.2% vs. 2.9%; *p = 0*.*002*). About 30% of the participants experienced severe fatigue; the proportion of persons reporting severe fatigue was 7-fold greater in those with PASC symptoms (Adjusted Prevalence Ratio [aPR] 6.73, 95%CI: 2.80–16.18). Persons with PASC symptoms were more likely to report poor quality of life (16% vs. 5%, *p<0*.*001*) and worse mental health functioning (Mean difference: -1.87 95%CI: -2.38, -1.37, *p<0*.*001*). Themes from in-depth interviews revealed PASC was experienced as debilitating.

**Conclusions:**

In this study, the prevalence of PASC among community-dwelling adults was substantial. Participants reported considerable coping difficulties, restrictions in everyday activities, invisibility of symptoms and experiences, and impediments to getting and receiving PASC care.

## Introduction

Over 493 million COVID-19 cases have occurred globally, 80 million in the US, resulting in over 6 million deaths globally and a million in the US [1]. COVID-19 survivors may experience post-acute sequelae (PASC). As the pandemic evolves, more research is needed on the presentation of PASC. The World Health Organization defines PASC as a condition occurring in individuals with a history of probable or confirmed SARS CoV-2 infection 3 months from the onset of COVID-19 lasting ≤2 months and unexplained by an alternative diagnosis” [2]. Common symptoms of PASC like fatigue, shortness of breath, and cognitive dysfunction, may be new-onset after an acute COVID-19 episode or persist from the initial illness, and they may fluctuate or relapse [2]. Common symptoms of PASC like fatigue, shortness of breath, and cognitive dysfunction, may be new-onset after an acute COVID-19 episode or persist from the initial illness, and they may fluctuate or relapse [2].

The burden of PASC is substantial [3], affecting an estimated 10–40% of persons with COVID [4–6]. Cardiac-related PASC is a subset of PASC, describing symptoms relating to the cardiovascular system, the burden of which is also substantial [7, 8]. Some proposed explanations to further elucidate the underlying mechanisms of PASC and cardiac PASC include inflammation [9], endothelial activation and microvascular thrombosis [10, 11], viral persistence [12], or triggering of latent viruses [13], impaired exercise metabolism [14], and profound cardiac deconditioning following viral infection [15]. The relationship between PASC and Cardiovascular Disease (CVD) and Cardiovascular (CV) risk factors has been observed to be bi-directional [16]. This was evident with CVD and CV risk factors being independent predictors for severe COVID-19 disease and death [17, 18]. On the other hand, previous infection with COVID-19 has been linked to increased risk of incident cardiovascular and cerebrovascular diseases and events [8].

These significant sequelae of COVID-19 have considerable implications for the health of persons with underlying multimorbidity, previously healthy patients, and communities previously at-risk for chronic diseases. These disparities have exacerbated COVID-19 risk and increased morbidity, disability, and death. We performed this convergent mixed-methods study to explore and understand experiences of PASC in a community-based cohort of people who tested positive for COVID-19.

## Materials and methods

### Study design

This was a convergent mixed methods design, with cross-sectional and qualitative descriptive method components. Convergent mixed methods involve collection and analyses of different but complementary data—quantitative and qualitative—and merging of these data for the purpose of comparing or combining the results [19]. Qualitative interviewing allowed for an understanding of the experiences of persons who had CVD and were considered at higher risk of severe COVID-19 symptoms and complications [20]. The quantitative arm assessed PASC symptoms that persisted after the acute phase of the infection.

### Participants and recruitment

Participants were recruited from a COVID-19 recruitment registry, a centralized database for people interested in COVID-19 studies at a large medical institution [21, 22]. This study included 18-year-old participants from the recruitment registry with a positive COVID-19. A total of 1,997 participants who were enrolled in the recruitment registry met eligibility requirements for the study. Participants were invited to join the study through automated invitations generated within Research Electronic Data Capture (REDCap), a HIPAA-compliant data management system. The COVID-19 recruitment registry has an overrepresentation of non-Hispanic White adults; thus, we used a stratified sampling approach with age and race/ethnicity strata to identify and recruit participants from the registry, with oversampling for persons who identified as American Indian or Alaska Native, Asian, Black, Hispanic or Latino/Latina adults [23, 24].

### Enrollment procedures

Baseline data were obtained between November 2021 and January 2022. The survey response rate was 70%; Out of 505 people who joined the study, 442 participants were included in the quantitative analysis. At survey completion, participants received a $10 digital gift card in compensation through an automated process in REDCap. In-depth qualitative interviews were also voluntary, if interested, participants completed an "interview interest form" inputting their availability. After receiving this form, study staff contacted participants to schedule and confirm the interview. After interviews were scheduled, participants were emailed their signed copy of the consent form with Zoom interview details. Participants unfamiliar with virtual meetings received technical support before the interview. From January 13 to February 4, 2022, in-depth interviews were conducted. Qualitative interview participants received $15 digital gift cards. S1 Fig in the Supplement shows a flow diagram of the participants; 442 people participated in the cross-sectional survey and 26 in the in-depth interviews.

### Quantitative study procedures and data collection

Data collection and reporting followed recommendations from the Consensus-Based Checklist for Reporting of Survey Studies (CROSS) S1 Table [25]. Surveys were self-administered via a secure electronic REDCapp database. The surveys asked about sociodemographic characteristics, COVID-19 testing, exposure, and vaccinations, pre-existing conditions (including CV risk factors such as hypertension and dyslipidemia; CVD such as coronary artery disease, heart failure, atrial fibrillation, chronic ischemic heart diseases, myocardial infarction, valvular heart disease; and other conditions such as chronic lung disease asthma, cancer, kidney disease, etc.).

#### PASC symptoms and quality of life

We developed PASC symptom questionnaires based on systematic reviews [26, 27]. These symptoms were categorized as by body systems. We assessed fatigue using the Functional Assessment of Chronic Illness Therapy (FACIT) Fatigue Scale (Version 4), a 13-item tool measuring levels of fatigue during usual daily activities. The scale uses a Likert scale (0–4, from “not at all fatigued” to “very much fatigued”); sum scores ranged from 0–52, and lower scores indicated severe fatigue [28]. We assessed quality of life, physical and mental health functioning using the PROMIS Scale Global Health (PROMIS-GH) v1.2 [29]. Items were scored on a Likert scale (1–5, “None” to “Very severe”) Higher scores indicated better global mental/physical health [30].

### Qualitative study procedures and data collection

Qualitative procedures followed Consolidated criteria for reporting qualitative research (COREQ) S2 Table [31]. Participants who reported a prior positive COVID-19 test and completed the surveys were eligible for the qualitative interviews. Among those who expressed interest, we purposely sampled those with and without underlying CVD or CV risk factors based on self-report. A sample size of 15–25 is typically sufficient for obtaining data saturation in qualitative studies, especially when the topic is highly focused on describing experiences on a specific topic [32]. We conducted remote in-depth interviews with 26 participants to reach thematic saturation. Before one-on-one interviews, participants gave oral, and written consent, and an electronic signature was obtained. The participants were emailed the consent form. In-depth interviews, lasting an average of 50 minutes, were recorded (audio only) and real-time notes were taken and stored in REDCap and on secured cloud storage. Both locations were on password-protected, encrypted servers. Quantitative data were collected using a patient-administered cross-sectional survey.

The research team developed a semi-structured interview guide with open-ended questions and probes based on PASC and COVID-19 literature [27, 33, 34]. The guide contained questions and probes to understand the experiences of people who had tested positive for COVID-19 with or without underlying CVD, who may be experiencing PASC, including those with CV risk factors. The guide asked about COVID-19 testing and acute infection symptoms. The interview guide for people without CVD or CV risk factors was modified to omit the first set of questions on CVD history, illness perception, self-management and health care utilization, use of telehealth during the pandemic, and impact of newly acquired CV complications. The rest of the questions and prompts were the same for people with and without CVD. These questions explored PASC experiences and impact on quality of life, with specific probes on fatigue, activity intolerance, care-seeking behaviors, and coping mechanisms. Each interview was conducted by a trained interviewer or notetaker.

### Open-ended survey responses

Data from the open-ended survey questions were compiled, imported into ATLAS.ti, and coded for newly emergent themes divergent from the interview data [35]. New themes and patterns that did not previous emerge from the in-depth interview data were combined into separate themes using inductive and deductive analysis techniques.

### Data integration and triangulation

We compared quantitative and qualitative data using an information matrix to determine convergence or divergence. We presented quantitative, qualitative, and integrated results. Applying both data and investigator triangulation techniques, we combined data from one-on-one interviews, direct observations during interviews, and the survey’s open-ended questions [36]. Methods used to ensure trustworthiness in the qualitative data collection and analysis [19, 37] included use of a mixed-methods approach, triangulation, interviewing techniques, iterative questioning, debriefing sessions with interviewers and note-takers, reflexivity commentary, highlighting study limitations and audit trail.

### Data analyses

In this convergent mixed-methods study, we analyzed quantitative and qualitative data simultaneously and combined the results [19]. We performed periodic quality assurance checks for the survey data. De-identified survey data were imported into Stata 16© for statistical analyses, interview transcripts and open-ended survey data were imported into ATLAS.ti for qualitative analyses.

#### Quantitative data analysis

Following a missing data analysis, the observations for the outcome variable determined to be missing at random (MAR) were excluded from the final analysis S1 Fig [38]. We examined the distribution of each outcome variable using descriptive statistics; means and standard deviations or medians (for interquartile range or range) for continuous variables, and frequencies, proportion/percentages for categorical variables. We summarized the sample characteristics to help identify the distributions, outliers, and missing data patterns. We also stratified these characteristics by report of PASC symptoms. We summarized the prevalence of report of current PASC symptoms, cardiac PASC symptoms, and new CVD diagnoses among the participants. We assessed the prevalence ratios of severe fatigue among persons who reported PASC symptoms, cardiac PASC symptoms, and new CVD diagnoses, compared to those without. We used generalized linear models with a Poisson distribution and a logarithmic link with linearized variance estimation to determine the prevalence ratios, and controlled for confounders in the fully adjusted models. Covariates included age, sex, race, educational, and income status. Quantitative analyses were performed in Stata I/C 16.1 [39].

#### Qualitative data analysis

All interviews were recorded, transcribed verbatim, and coded using ATLAS.ti Web and Desktop version 9 software [40, 41]. An inductive content analysis approach was applied toward analyses of the qualitative data. Transcripts were analyzed by research team members trained in qualitative data analyses; using both a deductive and an inductive approach and independent coding of two transcripts (5 research team members in total). We developed a codebook based on the initial codes and the interview guide [42]. The team met biweekly during this process to review and discuss the initial codes and codebook. The revised codebook was imported into the ATLAS.ti to guide the rest of the analyses. Using an iterative process, codes were merged into higher level subthemes that were later combined into larger themes. Themes were categorized and incorporated during data analysis and interpretation of the quantitative results.

#### Research team and reflexivity

The research and data analysis team consisted of persons from Black, White, and Arab backgrounds. Individual ideas regarding race/ethnicity, privilege, socio-economic status, clinical training, and public health practice, sex, gender, and expertise were discussed during data collection and analyses. The study team discussed and reflected upon these positionalities and possible influences on the analyses. Analytic memoing and frequent discussions were held with the research team, and an audit trail was maintained [19, 37].

### Ethics

The study received approval from Johns Hopkins Medicine Institutional Review Board (IRB00299548). All participants gave written consent, interview participants gave both oral and written consent and an electronic signature was obtained.

## Results

A total of 442 participants were included in this analysis. The mean (SD) age was 45.4 (16.2) years, 71% were female, 12% were Black/African American, 12% were hospitalized for COVID-19, with a mean (SD) length of hospital stay of 9.8 (17.5) days, 70% were employed and 33% were healthcare workers (Table 1). About 91% had received at least one dose of a COVID-19 vaccine–among them, 74% (296/400) had tested positive to COVID-19 before they received the first dose of the vaccine, and 58% had received a booster dose. Persons who reported PASC symptoms were older (mean age: 46.5 vs. 42.0; *p = 0*.*013)*, more likely to be female (74.3% vs. 61.2%; *p = 0*.*010*), have pre-existing conditions (49.6% vs. 34%; *p = 0*.*005*), and to have been hospitalized for COVID-19 (14.2% vs. 2.9%; *p = 0*.*002*).

**Table 1:** Sociodemographic characteristics stratified by report of PASC symptoms (N = 442).

Characteristics, M(±SD)/n(%)	Total, N = 442	PASC symptoms		*P*-value
Mean(SD)/n(%)		Yes (N = 339)	No (N = 103)	
**Age, years**	45.4 (16.2)	46.5 (16.2)	42.0 (15.7)	**0.013**
**Female** ^a^	311 (70.8)	251 (74.3)	63 (61.2)	**0.010**
**Underlying CV risk factors or CVD** ^b^	129 (29.2)	101 (29.8)	28 (27.2)	0.610
**Pre-existing conditions** ^c^	203 (45.9)	168 (49.6)	35 (34.0)	**0.005**
**Months since COVID-19 infection** ^d^	12.0 (5.3)	12.1 (5.4)	11.5 (5.1)	0.274
**Race/Ethnicity**				0.223
White	322 (72.9)	239 (70.5)	83 (80.6)	
Black/African American	55 (12.6)	48 (14.2)	7 (6.8)	
Asian	33 (7.5)	26 (7.7)	7 (6.8)	
Other	16 (3.6)	12 (3.5)	4 (3.5)	
Hispanic ethnicity^e^	31 (7.1)	26 (7.7)	5(4.9)	0.411
**Educational status**				0.353
High School diploma/≤GED	27 (6.1)	22 (6.5)	5 (4.9)	
Some college	100 (22.7)	81 (24.0)	19 (18.5)	
Bachelor’s degree	152 (34.5)	118 (34.9)	34 (33.0)	
Graduate degree	162 (36.7)	117 (34.6)	45 (43.7)	
**Household Income**				0.289
≤$39,999	64 (14.7)	52 (15.8)	11 (10.8)	
$40,000 –$69,999	68 (15.6)	55 (16.4)	13 (12.8)	
$70,000 –$99,999	77 (17.6)	61 (18.2)	16 (15.7)	
≥$100,000	183 (41.9)	131 (39.1)	52 (51.0)	
**Employed** ^f^	307 (69.6)	228 (68.3)	79 (77.5)	0.075
**COVID-19 hospitalization**				
Hospitalized, n (%)	51 (11.5)	48 (14.2)	3 (2.9)	**0.002**
Length of hospital stay, days, Median (IQR)	5 (2–10)	5 (3–11)	4 (2–10)	0.704
**COVID-19 Vaccination, n (%)**				
At least one dose^g^	400 (90.5)	307 (90.6)	93 (90.3)	0.714
Two doses	372 (84.2)	284 (83.8)	88 (85.4)	0.686
Booster dose	230 (57.6)	171 (55.9)	59 (63.4)	0.387
**Healthcare Worker** ^h^	146 (33.3)	110 (32.7)	36 (35.3)	0.631

Ref:

^a^Male;

^b^CV risk factors included hypertension and dyslipidemia; CVD included coronary artery disease, heart failure, atrial fibrillation, chronic ischemic heart diseases, myocardial infarction, valvular heart disease, etc.;

^c^Pre-existing conditions prior to COVID-19 infection (cardiovascular diseases, asthma, cancer, kidney disease, irritable bowel syndrome etc.);

^d^Median (IQR) months since infection was 12.4 (10.0–15.2) months;

^e^Non-Hispanic;

^f^Not employed;

^g^About 74% (296/400) of those who had received a COVID-19 vaccine had previously tested positive to COVID-19;

^h^Non-health care worker.

BMI: Body mass index. GED: General Education Development qualification.

Bold: Statistically significant at <0.05. P-values were estimated using t-tests for differences in means and chi-square test statistics for differences in proportions.

### Report of PASC and cardiac PASC

**Fig 1:**
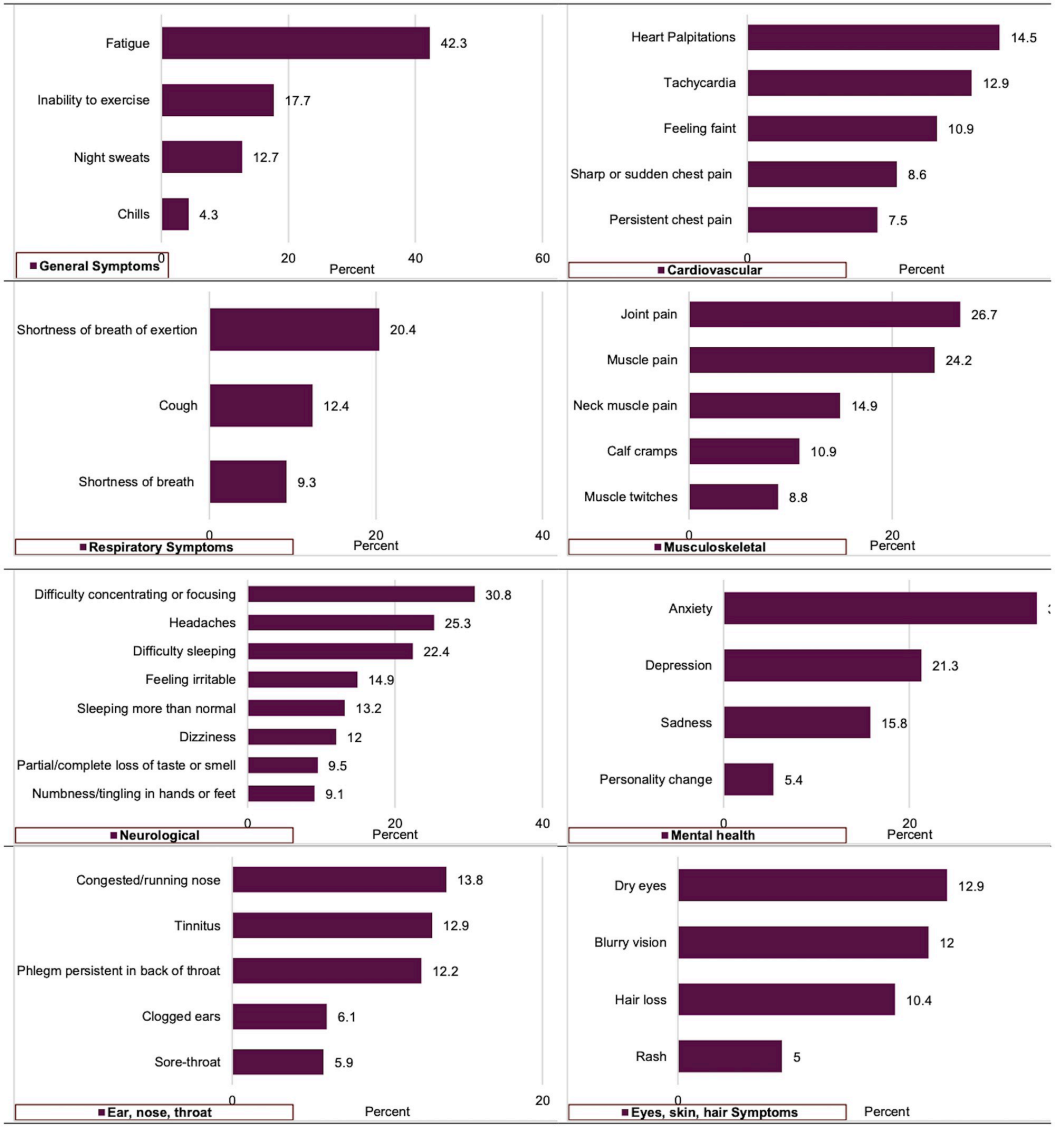
Reported Post-Acute Sequelae of SARS-CoV-2 Infection (PASC) symptoms (N = 442).

Among this sample of majority community-dwelling persons surveyed on average one year after testing positive for COVID-19, about 29% reported having pre-existing CV risk factors of CVD prior to COVID-19 infection, and 77% reported PASC symptoms at the time of survey administration; 43% reported current cardiac-related PASC symptoms and 27% reported newly diagnosed CV risk factors (such as hypertension, arrhythmias) or CVD (such as myocarditis, atrial fibrillation, heart failure). Fatigue (42.3%) and inability to exercise (17.7%) were the most frequently reported general symptoms (Fig 1); heart palpitations (14.5%), tachycardia (12.9%), and feeling faint (10.9%) were the most common cardiac-related symptoms (Fig 1). Other most common symptoms were shortness of breath (20.4%), joint and muscle pain (26.7%, 24.2%, respectively), difficulty concentrating or focusing (30.8%), headaches (25.3%), difficulty sleeping (22.4%), anxiety and depression (33.7% and 21.3%, respectively).

### Fatigue and quality of life

About 30% of the participants reported severe fatigue (FACIT-F scale), 28% reported moderate fatigue. PASC symptoms were associated with severe fatigue (Mean difference: -13.02 95%CI: -15.65, -10.38, *p*<0.001). In terms of ability to conduct everyday physical activities, about 17% and 8% reported moderate or little ability, respectively, to perform daily physical activities. Adjusting for age, sex, race, education, household income, and pre-existing conditions, the proportion of PASC patients reporting severe fatigue was 7-fold higher (Adjusted Prevalence Ratio [aPR] 6.73, 95%CI: 2.80–16.18). After adjusting for covariates, the proportion of people with severe fatigue was 4-fold and 2-fold higher in those with cardiac PASC symptoms and new CVD diagnoses, respectively (cardiac PASC: aPR 4.28, 95%CI: 2.94–6.22; new CVD diagnoses: aPR 2.32, 95%CI: 1.73–3.06). About 20% of the sample reported excellent quality of life, 34% very good, 33% good; 13% reported fair or poor. Persons with PASC symptoms reported worse quality of life (16% vs. 5%, *p<0*.*001*) and mental health functioning (Mean difference: -1.87 95%CI: -2.38, -1.37, *p<0*.*001*).

### Qualitative results from in-depth interviews

Sociodemographic characteristics of interview participants are shown in S3 Table. Mean age was 50, ranging 23 and 84 years). About 20% were men, 8% were Black or African American, 19% had been hospitalized, and 92% had received two doses of the COVID-19 vaccine.

#### Emergent qualitative themes

**Fig 2:**
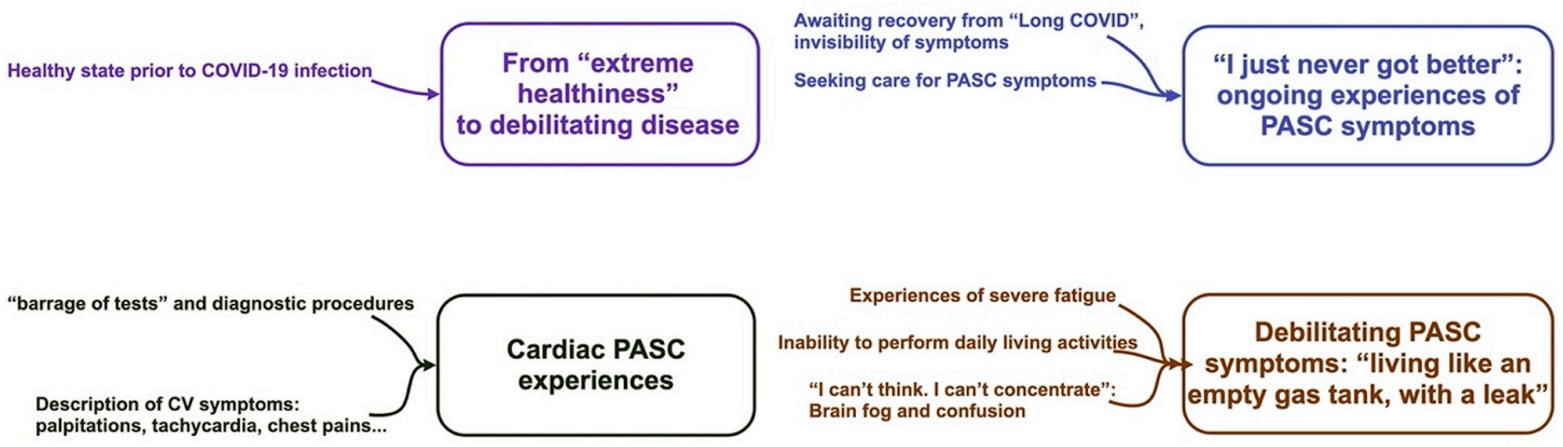
Graphical coding tree and emergent descriptive themes.

Four major themes emerged from the qualitative interviews (Fig 2), including: (1) From “extreme healthiness” to debilitating disease; (2) “I just never got better”: ongoing experiences of PASC symptoms; (3) Cardiac PASC experiences; (4) Debilitating PASC symptoms: *“living like an empty gas tank with a leak*.*”* Emergent themes, subthemes, and quotations are presented in S4 Table.

#### Theme one: From “extreme healthiness” to debilitating disease

Participants described their state of health and quality of life prior to COVID-19 infection. Some participants described their health as perfect; others discussed underlying diseases, including CVD that were diagnosed prior to March 2020. Participants compared their health before and after COVID-19 infection in interviews. According to one 62-year-old participant, they had “never been sick or missed a day of work due to any sickness,” but that changed after contracting COVID-19 infection (S4 Table, **Q1**).

#### Theme two: “I just never got better”: Ongoing experiences of PASC symptoms

We asked participants about lingering symptoms after COVID-19’s acute phase. Most interviewees continued to experience PASC symptoms (or "Long COVID"). Several participants hd conditions that may be related to COVID-19 infection or acute treatment. Furthermore, several participants were frustrated by symptoms’ invisibility and persistent debilitation. Some participants’ frustrations with PASC symptoms stemmed from a desire to look sick rather than healthy, so that their symptoms would be recognized by healthcare providers and others such as persons within their social network, work colleagues, etc. (S4 Table, **Q2**).

Some participants also described challenges regarding accessing care at "Long COVID" clinics. These difficulties included getting referrals, insurance coverage, high costs of care, care team dismissal of symptoms, and perceived inadequacy of PASC treatment modalities. In job loss and workers compensation situations, insurance restrictions and massive bills hampered access to PASC clinics. Several participants reported negative attitudes from care teams and social networks about their symptom reports, this included dismissal of their symptoms and calling PASC "psychosomatic". One participant said PASC clinics "weren’t particularly helpful" and turned to other chronic care practices and specialists, such as a myalgic encephalomyelitis/chronic fatigue [ME/CFS] specialists (S4 Table, **Q3**).

#### Theme three: Cardiac-related PASC

PASC symptoms may indicate organ and system damage. We asked about cardiac-related PASC symptoms, including newly diagnosed CVD. About half of the interviewees described ongoing cardiologist care or future plans to see one. Participants who contracted COVID-19 early in the pandemic discussed their confusion and frustration from lingering symptoms and no definite diagnosis from their team. They used other medical sources to confirm their symptoms. PASC symptoms can be non-specific, making diagnosis difficult, thus, initial diagnostic focus may be to rule out complications. Many of the participants reported undergoing a "barrage of diagnostic tests" without conclusive diagnoses. However, one participant described electrocardiography abnormalities few months after COVID-19 infection and new CVD diagnoses. (S4 Table, **Q4**).

In terms of cardiac-related PASC symptoms, persistent chest pain was common among interview participants. The characteristics and quality of the chest pain, as described, varied between participants. Resting heart palpitations and tachycardia were also described (S4 Table, **Q5**).

#### Theme four: Debilitating PASC symptoms

Severe fatigue was described as using the terms: "paralyzing" and "the worst fatigue ever." Participants said this affected daily performance (S4 Table, **Q6**). Inability to perform daily activities was a recurrent theme during the interviews. Participants listed regular activities made difficult by PASC (S4 Table, **Q7**). Most interviewees reported neurological symptoms. Many described "brain fog" and confusion and their daily impact. Participants frequently experienced amnesia and confusion (S4 Table, **Q8**). During interviews, the study team observed participant confusion. Participants appeared confused, lost their train of thought, or had trouble understanding questions.

### Integration of quantitative and qualitative results

Qualitative results complemented quantitative results. The integrated results were analyzed to show whether qualitative interview findings confirmed, expanded, or diverged from quantitative findings (Table 2).

**Table 2:** Integration and comparison of quantitative and qualitative results.

Domains	Quantitative Results	Qualitative findings	Mixed Methods meta-inferences
		Qualitative subcategories	
Baseline Health	46% reported pre-existing conditions, before COVID-19; while 29% reported pre-existing CV risk factors and CVD	1. From “*extreme healthiness*” to debilitating disease 1a. Healthy state prior to COVID-19 infection *“I’ve run half marathons*. *I’m a yogi*. *I used to be in the military*. *I was fit*, *active*…*Then I got COVID*, *and now*, *I’m on long-term disability*. *I have a walker*. *I have more diagnoses than I know what to do with”*– 44 year old woman	*Divergence* Some interview participants described perfect health, although prevalence of pre-existing conditions was high
Post-Acute Sequelae of COVID-19 (PASC)	77% reported PASC symptoms at enrollment, time since positive test was 12 months.	*2*. *“I just never got better”*: ongoing experiences of PASC symptoms 2a. Awaiting recovery from *“Long COVID*”, invisibility of symptoms *“All of a sudden*, *just like I came back down to the weakness and the fatigue and the body aches and the palpitations*, *and the heart—I’ve never recovered since then*. *I’ve never felt as good as I did that Friday before when I thought I could go back to work”– 44 year old woman* 2b. Seeking care for PASC symptoms “*if you can fix me*, *I’m going to try it”*: seeking alternative care *“I also went to integrative medicine and spent hundreds of dollars on herbs “– 44 year old woman*	*Confirmation* Participants’ description of continued experiences of PASC symptoms explains the high prevalence of PASC symptoms on the survey
Cardiac-related PASC	43% reported current cardiac-related PASC symptoms, proportion of persons reporting severe fatigue is 4-fold greater in persons who report PASC symptoms (95%CI: 2.94–6.22)	3. Cardiac PASC experiences 3a. Barrage of tests and diagnostic procedures *“They gave me a EKG*. *That was the first time I ever seen—it came back and said I had an incomplete right bundle branch block*. *That had never been reported before”– 60 year old woman* 3b. Description of CV symptoms: palpitations, tachycardia, chest pains… *“when I have heart palpitations*, *what happens is I can feel my heartbeat—I can feel my heartbeat very strongly and also see it very visibly throughout most of my body” –23 year old man*	*Expansion* Experiencing various possible cardiac-related symptoms and extensive CV assessments confirms the high prevalence of reported possible cardiac-related PASC symptoms
Fatigue and activity intolerance	30% were experiencing severe fatigue, proportion of persons reporting severe fatigue is 7-fold greater in persons who report PASC symptoms (95%CI: 2.80–16.18)	4. Debilitating PASC symptoms: *“living like an empty gas tank*, *with a leak”* 4a. Experiences of severe fatigue *“there’s bad days where I have to be served my dinner at the couch because I just don’t even have the energy to eat at the table…with my family”– 44 year old woman* 4b. Inability to perform daily living activities “*I just could not do anything*. *I couldn’t even jog two houses on my street*, *and I live on a flat street*. *It took me months and months to be able to do a jog at a very slow pace”– 60 year old woman* 4c. *“I can’t think*. *I can’t concentrate”*: Brain fog and confusion *“I got off the interstate*, *and I had no idea where I was… Sometimes I’ll be talkin’ and I’ll be like*, *“Wait*, *what*?*” I don’t even know what I was sayin’*. *My pills*, *I don’t know if I take my medicine*. *I don’t know*. *I don’t remember doing things that I do*, *like drivin’ home…I can’t think*. *I can’t concentrate*.*”– 41 year old woman*	*Confirmation* Understanding the severity of fatigue experienced after recovery and inhibitions in performance of daily activities explains the high prevalence of fatigue in persons experiencing PASC

CVD–Cardiovascular Diseases; CV–Cardiovascular; PASC–Post-Acute Sequelae of SARS-CoV-2 Infection; ECG–Electrocardiography

Themes on pre-COVID-19 health diverged from quantitative results of 46% with pre-existing conditions. Emergent themes and subthemes added depth to quantitative findings on PASC among community-dwelling adults. Results from open-ended survey questions are presented in S5 Table.

## Discussion

We studied PASC in a community-based cohort of COVID-19-positive people. This convergent mixed-methods study among COVID-19-positive community-dwelling adults supports several main findings. First, the prevalence of PASC symptoms was high among community-dwelling adults; second, the severity of the acute COVID-19 infection may not predict the experience of PASC symptoms; third, most reported general PASC symptoms were fatigue and possibly cardiac-related symptoms including heart palpitations, tachycardia, feeling faint, and chest pain; and fourth, PASC symptoms are debilitating and limit daily activities, diminishing quality of life and physical function. Quantitative data showed a high prevalence of PASC symptoms, including cardiac-related symptoms. PASC sufferers reported severe fatigue, lower quality of life, and mental impairment. Interviews revealed four themes: 1) from “extreme healthiness” to debilitating disease; (2) “I just never got better”: ongoing experiences of PASC symptoms; (3) cardiac PASC experiences; (4) debilitating PASC symptoms: “living like an empty gas tank with a leak.”

Prior studies reported similar PASC symptoms [5, 27, 43]. Fatigue (42.3%) was the most common symptom reported by participants. In people with cardiac PASC symptoms and new CVD diagnoses, severe fatigue was 4 times more common. Qualitative research added context to quantitatively reported fatigue. Participants’ descriptions of fatigue ranged from "living like an empty gas tank" to debilitating disease that prevented them from resuming normal activities after SARS-CoV-2 recovery. However, the impact of fatigue on instrumental activities of daily living was variable, with some participants continuing to work reduced hours or no longer able to work outside of the home. Fatigue was also more likely to be reported by persons with existing cardiovascular diagnoses. Nevertheless, it is evident that there was an association between reported cardiovascular symptoms and having a greater likelihood of having severe fatigue. These findings could be leveraged to improve care pathways for patients with CVD and COVID-19.

Our qualitative results on persistent symptoms and limitations in performing daily activities have also been reported in other PASC studies [44]. Inability to exercise was a common general symptom reported by participants (17.7%). Exercise limitations were ascribed to shortness of breath or tachycardia. None of the participants reported formal cardiopulmonary exercise testing (CPET). Reports of tachycardia were primarily based on commercial grade wrist actigraphy. A recent study reported that persistent dyspnea could not be explained by pulmonary, cardiac or ventilatory limitations post-acute COVID-19 among recovering patients who underwent CPET [45]. Further evaluations are needed to objectively determine COVID-19’s impact on exercise capacity. Determining if inability to exercise is due to deconditioning from prolonged illness or SAR-CoV-2 infection requires further research [15].

Early diagnosis and management of PASC may benefit patients with long-lasting symptoms. Due to the lack of a universal definition of PASC and validated treatment modalities, many patients are reluctant to access the healthcare system if their symptoms are not validated. PASC diagnosis and management require specific recommendations and policies. More investment is needed in under-resourced communities most affected by COVID-19 and PASC [46]. Cardiovascular diagnostic testing such as coronary computed tomography angiography, cardiac magnetic resonance imaging, and pulmonary function testing is needed to diagnose persistent cardiac sequelae. Unfortunately, these tests are often inaccessible to patients who do not reside near major medical centers. It is important that cardiovascular evaluation costs for PASC patients are covered by health insurance with no required cost sharing, similar to preventive services; otherwise, this could lead to additional barriers to care [46], particularly in under-served communities, further increasing the burden of cardiovascular disease and the negative impact of PASC. There are needs for transdisciplinary approaches to management, ongoing monitoring, and rehabilitation modalities at both population-level and individual-level interventions to promote recovery.

### Limitations and strengths

This study has several limitations. The surveys were online and self-administered, limiting enrollment to participants with moderate-to-high digital literacy. This study was based on self-report and thus subject to self-report or recall biases. It is unclear whether the symptoms and new diagnoses post-COVID were related to COVID, incidental or pre-existing prior to COVID-19. The exact timeline to the sequence of events may not be precise because participants depended on their memory for recall. In addition, although majority of the respondents who had received the COVID-19 vaccine tested positive to COVID-19 prior, there remains a possibility that participants who experienced post-vaccination side effects may have reported those as PASC symptoms.

This study has strengths despite its limitations. First, this study advances our understanding of PASC patients’ lives. The mixed-method design allows community-dwelling adults to report their PASC experiences. Qualitative data findings added rich nuances and perspectives to quantitative data interpretations. Both inductive and deductive research methods were used, and this study has accounts from mild, moderate, and critically ill hospitalized COVID patients. We also used data and investigator triangulation to cross-verify and validate qualitative findings.

## Conclusions

Our study highlights the high prevalence of PASC symptoms in community-dwelling adults who have tested positive for COVID-19. Persons who reported PASC symptoms reported severe fatigue, lower quality of life, and poor mental functioning. PASC symptoms can be debilitating and contribute to fear and hopelessness among persons experiencing these persistent symptoms. Despite activated coping strategies during the recovery process, significant challenges remain regarding receiving PASC care and achieving complete recovery.

## Supporting information

S1 Fig Study flow chart and participants inclusion. (TIF)

S1 Table Checklist for Reporting Of Survey Studies (CROSS) checklist. (DOCX)

S2 Table COnsolidated criteria for REporting Qualitative research (COREQ) checklist. (DOCX)

S3 Table Sociodemographic characteristics of interview participants (N = 26). (DOCX)

S4 Table Emergent themes and sub-themes from qualitative results. (DOCX)

S5 Table Themes from open-ended questions on survey data. (DOCX)
