# Supplementary figures and images for: “Living like an empty gas tank with a leak”: Mixed methods study on post-acute sequelae of COVID-19
Source: PLoS One. 2022 Dec 30;17(12):e0279684. doi: 10.1371/journal.pone.0279684 (PMC9803174; doi:10.1371/journal.pone.0279684)

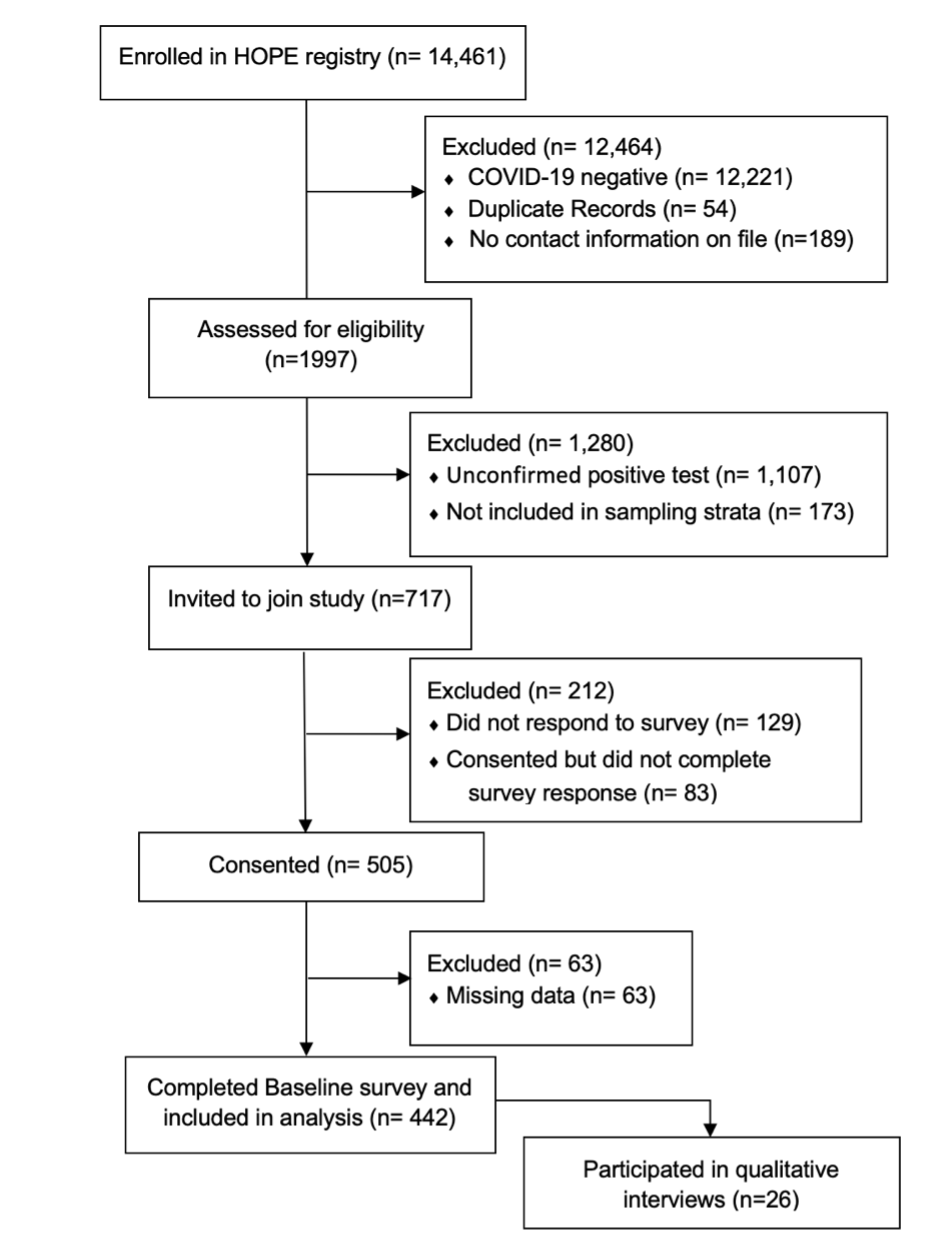

Supplement: S1 Fig — (TIF) [file pone.0279684.s001.tif]
