# Supplementary material for: “Living like an empty gas tank with a leak”: Mixed methods study on post-acute sequelae of COVID-19
Source: PLoS One. 2022 Dec 30;17(12):e0279684. doi: 10.1371/journal.pone.0279684 (PMC9803174; doi:10.1371/journal.pone.0279684)
Supplement: S3 Table — (DOCX) [file pone.0279684.s004.docx]

**S3 Table. Sociodemographic characteristics of interview participants (N = 26)**

| **Characteristics, M(±SD) / n(%)** | **Total (N = 26)** |
| --- | --- |
|  |  |
| **Age, years, M (±SD)** | 50.4 (16.7) |
| **Female, n (%)** | 13 (50.0) |
| **Race/Ethnicity, n (%)** |  |
| Black/African American | 2 (7.7) |
| Asian | 2 (7.7) |
| White | 20 (76.9) |
| Latino/Latina | 2 (7.7) |
| **Educational status, n (%)** |  |
| High School diploma/GED | 1 (3.8) |
| Some college/Associate | 5 (19.2) |
| Bachelor’s degree | 10 (38.5) |
| Graduate degree | 10 (38.5) |
| **Household Income, n (%)** |  |
| ≤$39,999 | 3 (11.5) |
| $40,000 – $69,999 | 5 (19.2) |
| $70,000 – $99,999 | 7 (26.9) |
| ≥$100,000 | 11 (42.3) |
| **Employed, n (%)** | 18 (75) |
| **COVID-19 hospitalization** |  |
| Hospitalized**, n (%)** | 5 (19.2) |
| Length of hospital stay**, M (±SD)** | 18.2 (16.9) |
| **COVID-19 Vaccination, n (%)** |  |
| At least one dose | 26 (100) |
| Two doses | 24 (92.3) |
| Booster dose | 16 (61.5) |
| **Healthcare Worker, n(%)** | 6 (23.1) |
| **Pre-existing Cardiovascular Risk Factors/Conditions, n(%)** |  |
| Hypertension | 5 (19.2) |
| High Cholesterol | 6 (23.1) |
| Ventricular Arrhythmias | 3 (11.5) |
| Valvular Heart disease | 2 (7.7) |
