# Supplementary material for: “Living like an empty gas tank with a leak”: Mixed methods study on post-acute sequelae of COVID-19
Source: PLoS One. 2022 Dec 30;17(12):e0279684. doi: 10.1371/journal.pone.0279684 (PMC9803174; doi:10.1371/journal.pone.0279684)
Supplement: S4 Table — (DOCX) [file pone.0279684.s005.docx]

**S4 Table. Emergent themes and sub-themes from qualitative results**

| **Themes** | **Sub-Themes** | **Sample Quotations** |
| --- | --- | --- |
| 1. From “*extreme healthiness*” to debilitating disease | 1a. Healthy state prior to COVID-19 infection | *Q1. “One of the reasons I was upset, extremely upset about having COVID was it was the first day of work I’ve ever missed in my life due to illness ever, ever, and I’m 62. You would need more than just a sniffle to keep me—I go to work ‘cause I just don’t get sick... I’m never sick, and I never stay home because I’m sick. COVID was a lot to deal with real quick.”* - 62 year old man |
| 1. *“I just never got better”*: ongoing experiences of PASC symptoms | 2a. Awaiting recovery from *“Long COVID*”, invisibility of symptoms | Q2. *“my family being [un]able to see [the] change… people look at me, and I don't look sick. Even my doctors are like, "You look healthy," and I'm like, "I know," but I almost wish I looked sick, so some people wouldn't look at me crazy when I have to sit and rest and I'm out of breath walking somewhere or whatever because I look like a youngish, relatively healthy person. I am not.- 44 year old woman* |
|  | 2b. Seeking care for PASC symptoms | Q3. “*I belong to three post-COVID clinics…and they have not been particularly helpful beyond the initial intake and general analysis. These are mostly pulmonologists who are used to seeing very sick pulmonology issues. I have gotten the vast majority of my actual care from a ME chronic fatigue specialist, an internist in California*.” *- 54 year old man* |
| 1. Cardiac PASC experiences | 3a. “*barrage of tests*” and diagnostic procedures | Q4. “*I know that I was scheduling lots of tests, they tried to figure things out. What they did notice is that my EKG, it was kind of abnormal before, but now there was move involved with it. At the end there was a new [T] wave inversion, and they put me in the hospital for a day just to monitor things, and just they even noticed visually I looked tired all the time. Even now, still feeling pretty sluggish a lot. I have high blood pressure now, never did before*.” *- 33 year old man* |
|  | 3b. Description of CV symptoms: palpitations, tachycardia, chest pains… | Q5. *“I was having pretty bad heart palpitations for somebody who just walked up the stairs. I would go from sitting to standing and my heart would start pounding. I would walk up or down a flight of stairs and my heart would start pounding” - 23 year old man* |
| 1. Debilitating PASC symptoms: *“living like an empty gas tank, with a leak”* | 4a. Experiences of severe fatigue | *Q*6*.“ I'm like a car that now has had its thing punctured and I'm just I can't do anymore." Cause unfortunately I feel like a gas tank that now exists with a whole hole in it” - 26 year old woman* |
|  | 4b. Inability to perform daily living activities | Q7. *“I've never experienced fatigue like this. This isn't something that makes sense because, like I said, I was in the military, a long-distance runner. This fatigue I cannot push through. I can't push through it. I wake up feeling like I've been awake for days. If I do too much, I'm in bed for two days, cannot do anything” - 44 year old woman* |
|  | 4c. *“I can’t think. I can’t concentrate”:* Brain fog and confusion | Q8. *“I have brain fog, and I was in therapy for a cognitive decline, which I have. My brain just doesn't work the way it used to, and so I can't—I have a hard time keeping track of, I guess, my train of thought and where I was supposed to go on that train” - 44 year old woman* |
| ME: myalgic encephalomyelitis | | |
