# Supplementary material for: “Living like an empty gas tank with a leak”: Mixed methods study on post-acute sequelae of COVID-19
Source: PLoS One. 2022 Dec 30;17(12):e0279684. doi: 10.1371/journal.pone.0279684 (PMC9803174; doi:10.1371/journal.pone.0279684)
Supplement: S5 Table — (DOCX) [file pone.0279684.s006.docx]

**S5 Table. Themes from open-ended questions on survey data**

| **Sub-Themes** | ***Sample Quotations*** | **Comparison with in-depth interview themes and Quantitative data** |
| --- | --- | --- |
| **Divergence** | | |
| Uncertainty about link between possible PASC symptoms and COVID-19 | *“Not sure if these are from COVID or my arthritis and allergies.” – 58 year old man*  *“I have also never experienced any symptoms in this nature and have not fully recovered since. I'm not sure I will ever be "normal" again” – 37 year old woman* | Majority of interview participants expressed uncertainty regarding link or direct association between COVID-19 infection and “PASC” symptoms |
| **Expansion** | | |
| “*feeling lucky”* for no experience of PASC | *“I feel lucky that I don't have lasting symptoms from COVID” – 73 year old woman* | Some interview participants expressed feeling of well-being and no experience of PASC after recovery from acute infection |
| Debilitating PASC symptoms | *“I am a shell of who I once was… I have had severe leg muscle & joint pain since, so much so that the pain pump doesn't help…I'd like to know when the continuing issues I have will be over? When will life get back to normal?” – 53 year old man*  *“Have dealt with long haul covid for more than a year now. it has been debilitating and kept me out of work and unable to care for myself or my kids”37 year old woman*  *“Brain fog is severe…I have experienced constant long COVID symptoms every minute, of everyday for eleven months now” – 54 year old man*  *“I feel like everything weighs twice as much, like stairs are twice as high (each step) and like I'm underwater. My heart feels bruised.” – 47 year old woman* | Similar to results from interview and quantitative data, participants described PASC symptoms as related to COVID-19 infection as debilitating |
| Exacerbation of underlying conditions | *“Considered a long hauler. It's been almost 2 years … I cannot focus or remember things. I never had this before COVID-19. I started wheezing again, I haven’t been wheezing in years” – 33 year old woman* | Interview participants described PASC symptoms as devastating and possibly exacerbating symptoms of pre-existing conditions |
| **Convergence** | | |
| New diagnoses of conditions post-COVID | *“I have 40-60 percent heart blockage I didn't have before COVID-19 diagnosed in 2020” – 50 year old woman*  *“I have been diagnosed with AfIB, and now take blood thinner. I was in ER several times for Afib. They cardioverted me once for it” – 51 year old man* | Interview participants discussed new diagnoses of chronic conditions |
| COVID-19 re-infections and PASC | *“I have had COVID twice now and the long lasting symptoms have been a bit worse for the second time than the first but all the symptoms are similar” – 24 year old woman* | Some interview participants also reported more than one COVID-19 re-infection episodes |
| Resignation, hopelessness, and searching for meaning | *This illness is so dejecting. I read articles of people committing suicide, and I totally get it. All of my diagnostic tests have been 100% normal. I've seen 11 different physicians of varying specialties and been dismissed by all of them. My primary care doc specifically told me not to make a follow-up appointment... I am a shell of who I once was. The mental and emotional toll far overshadows the physical toll” – 36 year old woman*  *“Long Covid has completely altered my life. My pain and psychological side effects never let up. They are so bad that I had to resign my position as the Director of a software company and take a lesser role…I have lost faith that I will return to normal” – 54 year old man* | This further confirms expression of hopelessness, symptoms report dismissal, and mental/emotional impact of living with PASC results from in-depth interviews and quantitative data |
